# A Serial-Mediation Model to Link Entrepreneurship Education and Green Entrepreneurial Behavior: Application of Resource-Based View and Flow Theory

**DOI:** 10.3390/ijerph18020550

**Published:** 2021-01-11

**Authors:** Irfan Hameed, Umer Zaman, Idrees Waris, Owais Shafique

**Affiliations:** 1 College of Business Management, Institute of Business Management, Karachi 75190, Pakistan; irfanhameed.iu@gmail.com; 2 Endicott College of International Studies, Woosong University, Daejeon 34606, Korea; 3 Faculty of Management Sciences, University of Turbat, Turbat 92600, Pakistan; idress1988@gmail.com; 4 Instituut of Business, Management and Administrative Sciences, The Islamia University of Bahawalpur, Bahawalpur 63100, Pakistan; owais.shafique@iub.edu.pk

**Keywords:** entrepreneurship education, resource-based view, flow theory, commitment to the environment, university entrepreneurial support, environmental sustainability, environmental motivation, green entrepreneurial behavior

## Abstract

Eco-conscious behaviors have become a global imperative for entrepreneurs, as the consumer demand for products and services has become increasingly green. Hence, the purpose of this study is to identify the role of entrepreneurship education in environmental sustainability as measured by the launch of green ventures. This study also aims at extending the literature of resource-based view and flow theory by highlighting their application into the green venturing context. Data were collected from 420 Pakistani students having studied an entrepreneurship course in their university life by using the convenience sampling technique. The covariance based structural equation modeling (CB-SEM) was used to test the hypothesized relationships, and it was identified that entrepreneurship education evokes commitment to the environment, subsequently leading towards university green entrepreneurial support, environmental motivation, and green entrepreneurial behavior. University green entrepreneurial support also significantly influences green venturing; however, environmental motivation does not affect green entrepreneurial behavior. The findings of this study can be useful for policymakers in higher educational institutions, regulatory bodies, and diverse-government agencies dealing with UN sustainable development goals.

## 1. Introduction

The fastest-growing green market has been projected to reach USD 2.74 trillion by 2020 [1], creating significant opportunities for new and existing entrepreneurs, as well as challenges for the marketers and policymakers to address issues of global concern (e.g., deforestation, air pollution, food and water insecurity, biodiversity loss, and global warming) [2]. Despite the uncertainties and varied opinions on the rate of global warming, environmental scientists are positing that the human race is experiencing unusual rapid global warming due to unsustainable human activities [3]. The elevated levels of greenhouse gases in the environment (e.g., carbon dioxide, water vapor, methane, chlorofluorocarbons, and nitrous oxide) have created a global concern about environmental degradation, as it has come to a point where it is being considered a threat to the human existence [4].

Despite growing international pressure, the developing nations have been accused of lack of seriousness towards the environment, mismanagement of their natural resources, as well as ignorance towards ecological damage. The government of Pakistan has legislated the Pakistan Climate Change Act [5] to meet the nation’s obligations under the international conventions relating to climate change and to address the effects of climate change. Despite various efforts exerted by the Pakistani government to combat climate change, it has still ranked 169 out of 180 counties around the world in the Environmental Performance Index [6], due to its incapability to meet the performance on environmental health and ecosystem vitality. To overcome these issues, the Government of Pakistan launched The Clean Green Pakistan Index (CGPI) in 2019 [7] under the Ministry of Climate Change (MoCC) that motivates cities to improve neighborhood conditions. Moreover, the National Youth Development Framework was developed in 2019 having a strategic focus of “achieving sustainable development goals” by ensuring access to technical, vocational, and civic education to 64% youth in Pakistan [8]. Concerning this, the United Nations Environment Programme [9] posited the importance of a green economy to improve the quality of life and establish sustainable development. Furthermore, the National Incubation Center was launched in Pakistan in February 2020 by the Ministry of Science and Technology to empower people to initiate their startups [10]. This clearly states the level of seriousness in the power corridors of Pakistan. Through these initiatives, the government will provide low-cost loans to the youth (agreed by UNDP, ages from 21–45 years) for the establishment of small business enterprises [11].

In this regard, small-medium enterprises have a paramount role as they contribute $ 86 billion to the GDP of Pakistan. SMEs have 35% value addition and out of 3.2 million enterprises, 99% are SMEs. The contribution of SMEs is vital in all major sectors of the economy e.g., GDP 30%, exports 25%, and industrial employment 78% [12]. Thus, SMEs are directly contributing to the economic growth and development of Pakistan. The potential role of university education will promote the launch of green ventures in the country and maximize the share of SMEs in the growth of an economy. Entrepreneurial behavior refers to the amalgamation of ideas, capital, and resources, along with the element of creativity and empowerment [13]. Through innovative ideas, green entrepreneurs around the globe have launched green products that meet individual health and environmental standards. Particularly, the young generation is more motivated to contribute to the betterment of society through green entrepreneurial ventures [14]. In the last decade, entrepreneurs have become instrumental in solving persistent environmental problems to societal changes through social innovation, technology, and green entrepreneurship [15,16]. Most of the green entrepreneurs organize businesses resulting in doing activities like recycling, energy efficiency, sustainable mobility, organic agriculture, and renewable energy [16]. Despite the launch of green businesses around the globe, small and medium enterprises (SME) still consider green businesses as something new [17]. Similarly, Omar and Samuel [18] posited that large manufacturing businesses are more concerned about active sustainable management than SMEs. Past studies have revealed that low awareness regarding environmental issues is one of the main obstacles SMEs face when trying to adopt sustainable management [19]. The Environment Agency [20] surveyed over 1000 UK SMEs, the survey revealed that more than 86% of the participants did not believe that they could contribute to environmental sustainability. The survey concluded that SME’s lack of awareness regarding environmental sustainability is one of the reasons that hinder them to participate in environmental management activities.

Extant literature depicts that limited attention has been paid to university education’s role on student’s green entrepreneurship behavior; specifically, on the impact of entrepreneurship education from the perspective of social activity creating employment opportunities has been not sufficiently analyzed. As part of a global trend, universities are transforming from teaching and research to assuming a third role in sustainable economic development [21,22]. The large-scale societal transitions towards sustainability are considered to be fundamental knowledge-driven [23]. The external environment can play a pivotal role in universities’ decision making regarding various activities. Universities actively scan their environment and adopt the worthwhile latest trends. Similarly, universities are taking part in awareness programs and promoting green entrepreneurship at their respective university’ campuses. However, there is an important question to be answered, whether entrepreneurship can be engrained through education. Inconsistent viewpoints of authors have been observed. Prior meta-analyses indicate a positive relationship between entrepreneurship education and entrepreneurial activity [24,25]. Researchers reported a positive impact of entrepreneurship education in setting up a new business [26,27,28].

There are two hundred and eighteen recognized universities and degree awarding institutions (DAI’s) in Pakistan as per Higher Education Commission. Two-hundred deal in more than one discipline including business education, engineering and technology, medical, etc. The remaining eighteen deal with any of the specific disciplines of education. Eighty-four universities/DAI’s belong to the private sector and one hundred and thirty-four are public sector (Higher Education Commission [29]. The economic contribution of higher education institutions is important as the main role of HEIs is to promote innovation so that the solutions to global challenges, for instance, environmental protection, resource security, healthcare, international relationships and development can be rectified. Additionally, the higher education sector is a natural partner to the knowledge-based economy. As is the source of advanced learning and new information from research, universities help in training the students of tomorrow while supporting the innovations of today [30]. Moreover, previous research works depict the positive impact of higher education institutions on sustainable development [31].

A recent study by Yi [32] explored the role of universities in promoting green entrepreneurship by merging the idea of environmental values with academics. Additionally, universities provide training facilities to the students to fulfill the requirements of current needs and support them to launch green businesses after graduation [33]. In this regard, Rothaermel, Agung, and Jiang [34] pointed out the importance of two key factors; first is the role of organization, second is the role of support system for the start-ups of green businesses. The first factor is related to the support of universities in creating green business ideas, providing the green incubation. The second factor is related to the provision of entrepreneurial education and providing support for ecological values. Ginanjar [35] studied higher education students’ entrepreneurial mindset and found the positive influence of entrepreneurial education on the development of students’ entrepreneurial creativity. Concerning green entrepreneurship, Demirel, Rentocchini, and Tamvada [36] found that green entrepreneurship support helps students to form positive perceptions regarding green initiatives.

Further, Matlay et al. [37] corroborated an optimistic view of teaching entrepreneurship courses by mentioning a significant relationship between venture creation and entrepreneurship education, unfolded that it embeds skills, knowledge, and a better entrepreneurial attitude in the students. On the other hand, some studies revealed the insignificant effect of entrepreneurship education on entrepreneurial intention [38,39]. There is no final verdict from researchers on a suitable conceptual model assessing the contribution of entrepreneurial education to individuals’ green entrepreneurial behavior. Therefore, Martin, McNally, and Kay [25] described that a better understanding of behavioral characteristics will enable researchers to understand the entrepreneurial instinct of an individual. Thus, it can be conceptualized that green entrepreneurship behavior is not merely a business, but a social activity aiming to protect and preserve the natural environment [40].

Therefore, this study has combined the resource-based view and flow theory approach to predict the influence of green entrepreneurship determinants on university students’ green entrepreneurship behavior. The resource-based view suggests the outcome is based upon the available resources. The external resource, university entrepreneurial support, and internal capabilities of the student, i.e., commitment to the environment and entrepreneurial motivation have been studied. The consideration sphere also specifies the role of education in motivating students to be green entrepreneurs, following the motivation to behavior approach of flow theory. While considering the university support and student’s commitment towards the environment as enablers infusing green entrepreneurship behavior in university graduates of Pakistan.

## 2. Literature Review

### 2.1. Theories Grounding the Study

This study used an integrated framework of Resource-Based View Theory (RBV) and Flow Theory (FT). The intervening relationships leading to green entrepreneurial behavior have been conceptualized based on the above-mentioned theories, where universities’ green entrepreneurial support was extracted from RBV and FT built the basis for entrepreneurial motivation.

#### 2.1.1. Resource-Based View Theory

“I am not asking what determines whether a particular firm can grow, but rather the very different question: assuming that some firms can grow, what principles will then govern their growth, and how fast and how long can they grow?” [41]. Penrose presented the ground-breaking work on the Resource-Based View (RBV) and stressed the importance of internal resources for the growth of the firm. Lack of resources creates obstacles in the firm’s growth. RBV stresses the internal resources to develop a competitive advantage [42]. Resource means anything attached to the firm in the form of strength or weakness. These can be tangible or intangible linked with the firm semi-permanently. The examples are in-house knowledge of technology, machinery, brand names, employment of skilled personnel, efficient procedures, capital, and trade contacts. [43]. The resources are combinations of all the facilities that can help in the development of value creation strategies. These include assets, capabilities, processes, knowledge information, and attributes. However, the three factors widely stressed in the literature are; physical, human, and organizational capital resources [42].

The university green entrepreneurial supports are related to the green entrepreneurial processes and behaviors, including the university’s actors, institutional settings, and resources. This can be inferred with reference to the concept of university support systems [43] and entrepreneurship theories [44]. Similarly, Yi [32] has used green university entrepreneurial support to implement the argument of Timmons [45] that resource-based theory holds that entrepreneurial resources are vital factors in the survival and development of start-ups. Being a successful entrepreneur requires a combination of resources. The availability of financial resources can help obtain many other resources, i.e., up-to-date systems/technology, market knowledge, infrastructure, and information regarding opportunities and trends [44]. Universities being the hierarchal organizations have the access to the resources and have developed incubation centers for the support of its graduating students. These incubation centers provide students with the necessary resources to start and operate the business at the initial stages [46]. Further, universities help students to develop market-based competencies and resources to step into the market. They provide them office space, telephone, internet, and connections with the industry in general and seed money for business launch in particular [47]. The current research study is an effort to link the RBV with sustainable entrepreneurship behavior, where resources can be provided by the universities to the aspiring entrepreneurs. This availability of the resources related to the launch of green ventures has been used as “university green entrepreneurial support” in this study.

#### 2.1.2. Flow Theory

The Flow Theory has been defined as “the state in which people are so intensely involved in an activity that nothing else seems to matter; the experience itself is so enjoyable that people will do it even at great cost, for the sheer sake of doing it.” [48].

Csikszentmihalyi originally presented the flow theory in 1975 as a mean to understand the motivation. It is considered as a psychological state explaining the peak feeling of the cognitively efficient, motivated, and happy individuals [49]. The integration of motivation, personality, and subjective experience has a desirable outcome, as suggested in flow theory. The importance of the phenomenon can be gauged with its application in various fields, with recent implications on the students learning [50]. In the early phase, this theory was applied to understand the best condition of the sportsmen. The implications of FT are even broader and diverse than its application in sports. It has also been used in leisure, users’ flow experience in human-computer interaction, computer-mediated technologies [51] and to comprehend navigation behaviors in online environments [52].

Flow theory provides an essential direction to probe intrinsic motivation. Liao [50] in his study on learner motivation suggests, “Flow is an intrinsic motivation”. Self-motivated learning is one of the best ways to learn, and the flow state is an intrinsic motivation, which can stimulate users to do an activity with inner joy. Motivation has two major divisions: intrinsic and extrinsic motivation. In extrinsic motivation, a favorable outcome is anticipated in the form of a reward, and intrinsic motivation is the willingness to act without the hope of a benefit particularly associated with that action [53]. This motivation can be translated into a relevant behavior by the encountering individual. Flow experience has four dominant features: intrinsic interest, feeling in control, feeling curiosity, and focusing attention. Trevino and Webster [54] found in their study that flow resulted in high effectiveness and a positive attitude. Chan and Ahern [55] postulated that the goal of education is to gain knowledge/skills and students can be best motivated to learn in an educational setting. Intrinsic motivation will help them to learn and apply the concepts practically. It has been believed that the good quality of systems and education can alone motivate students towards particular behavioral outcomes [56].

Environmentally motivated students will be actively looking for opportunities to start new green ventures [57]. The students can be equipped with entrepreneurial skills during university education, which can be translated into entrepreneurship behavior [58]. Furthermore, the current study aims at extending the application of flow theory in green entrepreneurship behavior. A major focus on the importance of green business will thus motivate them to start green ventures, which can also be referred to as green entrepreneurial behavior.

### 2.2. Conceptual Model

#### 2.2.1. Entrepreneurship Education and Commitment to the Environment

Entrepreneurial education programs are considered efficient means that provide the required skills and knowledge to aspiring entrepreneurs [27,59]. Concerning the significance of entrepreneurial education, researchers have expressed confidence over entrepreneurial education regarding a paradigm transfer in teaching rationale as well as in learning for a sustainable world [60]. Past research works have further highlighted the importance of environmental concern shaping individual entrepreneurial behavior [61]. Universities groom students’ world views and ecological values by formulating appropriate curricula and course structure which transform into environmental commitment [62]. Further, researchers argued that the new generation is more committed towards the safety of the ecosystem [63], and demonstrates their commitment to launch green ventures. Rothaermel, Agung, and Jiang [34] pointed out the significance of organizational support for entrepreneurial education leading to ecological values. Thus, it is appropriate to assume that entrepreneurial education fosters increased commitment towards the safety of the environment. Hence, we postulate that:

**Hypothesis 1 (H1):**  *Entrepreneurial education has a significant positive influence on the commitment to the environment.*

#### 2.2.2. The Role of Commitment to the Environment

Researchers argued that commitment is the psychological attachment with environmental issues [64]. Commitment to the environment is a competitive edge of the organizations as it builds a positive image of the company [65]. The commitment influences entrepreneurial behavior including management style, production, and other relevant areas of commencing the business [66,67]. Universities committed to the environment devise their programs towards the wellbeing of society.

Preedy, Glatter, and Levačić [68] highlighted three perspectives on the change process in educational institutions. These are technological, political, and cultural. The cultural perspective is concerned with the social setting, it establishes a commitment to everyday reality. In educational terms, this may be interpreted as the need to update practices in keeping with the findings of international research, and to continually conform to national trends [69]. Educational changes occur at different levels, the systemic, university and classroom level [70]. Furthermore, alterations in staff-student relationships from teacher-centered to student-centered create the need for modification of teaching practices, policies and procedures [71]. In order to cope up with the changing needs of the various stakeholders, universities devise their programs accordingly. One of the major initiatives of the universities is the introduction of the incubation centers within the universities [72]. The students having this ability to think ecologically can identify ways to communicate with the university regarding the requirement for the launch of green enterprise [64]. Thus, individuals with more commitment towards the environment can influence the management of the university to launch green support programs. Additionally, students’ environmental commitment affects higher education institutions to provide support for the startup of green enterprises. Hence, it can be hypothesized as follows:

**Hypothesis 2 (H2):**  *Commitment to the environment has a significant positive influence on university green environmental support.*

Suasana and Ekawati [73] postulated that the higher commitment of an entrepreneur leads to the safeguard of the environment. Students’ commitment to the environment develop an urge to find ways for constructive deeds transforming it as their internal motivation [74]. Highly environmentally committed students grow this internal motive to work for the betterment of the environment. Thus, it is hypothesized that:

**Hypothesis 3 (H3):**  *Commitment to the environment has a significant positive influence on entrepreneurial motivation.*

Committed environmentalists develop the tendency of doing constructive acts rather than just watch the environmental degradation. Their commitment forces them to play their part in the economy [75]. They develop a feeling of concern and try to find solutions to this situation. This feeling results in the desire to launch sustainable enterprises for safeguarding the environment. Bergmann, Hundt, and Sternberg [76] also highlighted the role of individual features in the commencement of green entrepreneurial activities. Therefore, it is proposed that:

**Hypothesis 4 (H4):**  *Commitment to the environment has a significant positive influence on green entrepreneurial behavior.*

#### 2.2.3. The Influence of University Entrepreneurial Support (UES)

Researchers pointed out the importance of university support in the creation of new businesses [76,77]. The literature supports the university’s inclination to fostering ecological values into students through green entrepreneurial support systems, or activities that promote green entrepreneurship. Furthermore, studies have applied multilevel strategies to address the effects of individual characteristics, university support programs, and institutional environment on entrepreneurial intention among students [78]. University experiential- learning programs provide a hands-on experience to students embedding skills and influence students for venture creation [79]. The concept of entrepreneurial university assumes diverse perspectives, but there is consensus on the importance of supporting the education of entrepreneurs and an environment conducive to innovation and entrepreneurship [76]. The influence of university support may direct them to participate in entrepreneurial activities [78].

Wagner et al. [79] posit that the university support system is essential for sustainable development in the region. According to them, the support system consists of providing incubators activities, entrepreneurial knowledge and services for green start-ups. With respect to the affirmative role of educational institutions, Fichter and Tiemann [43] supported the role of entrepreneurial education in green business activities. Other researchers identified that sustainable business development depends upon individual knowledge, competencies, attitude, and skills to manage sustainable businesses [80].

The university support is not only limited to providing entrepreneurship courses, it has a significant role in the development of self-efficacy in starting a new venture [81]. In addition to this, universities provide opportunities to interact with entrepreneurs that serve as a role model, design business plans, nurture business skills, and awards to innovative business ideas [82]. Recently, several studies have demonstrated the role of perceived university support in the creation of new business ventures [78,83]. Since a university that supports green entrepreneurial activity has a positive impact on green entrepreneurship behavior. Therefore, we drive the following hypothesis:

**Hypothesis 5 (H5):**  *University green entrepreneurial support has a significant positive influence on green entrepreneurial behavior.*

#### 2.2.4. Entrepreneurial Motivations (ENM)

Individual motivation is a major factor that helps and imparts skills to start a new venture [84]. The motivation of entrepreneurs drives them towards strategy formulation and engaging in the entrepreneurial activities that contribute towards the launch of sustainable ventures [85,86]. In an entrepreneurial context, it is the motivation and the perception of individuals that support the entrepreneurial initiatives [87]. Motivation can modify individual behavior and stimulates a person for the creation of a new venture as a career choice [63]. It has been observed that when an individual is positively motivated by personal needs, he or she will strive hard to obtain the outcomes (i.e., commitment to a sustainable environment). Moreover, Rekha, Ramesh, and Jaya-Bharathi [88] explained that creativity means innovative actions, since the human mind cannot live without this, so entrepreneurial actions of creating solutions to problems are the imperative function which is not simply a function of doing something new [89]. Robichaud, McGraw, and Alain [66] denoted entrepreneurial motivations as one of the major factors that help to create a business venture. These motives influence entrepreneurial behavior including management style, production, and other relevant areas of commencing the business [66,67]. Motivation must be a component of an entrepreneur’s personality [90]. The studies depict the significance of self-motivation for the launch of new business ventures. Based on the above arguments, we can assume that entrepreneurial motivation leads to green entrepreneurial behavior. Hence, we hypothesized that:

**Hypothesis 6 (H6):**  *Entrepreneurial motivation has a significant positive influence on green entrepreneurship behavior.*

**Figure 1:**
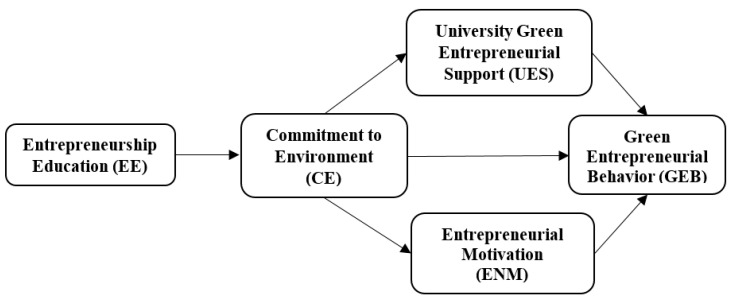
Conceptual Model.

Figure 1 represents the conceptual model of the study. The hypothesized relationships between the variables have been presented in a pictorial form in the figure.

## 3. Materials and Methods

### 3.1. Sampling

The unit of analysis was university students having studied the formal course of Entrepreneurship. The students and recent graduates of different business universities of Sindh (a province of Pakistan) were approached through an online survey. On the title page, the reason for sending the questionnaire was explained along with the option to quit at any point of time during the survey if they feel uncomfortable. They were assured that their identity will be kept confidential and will not be made available to anyone at any cost. The questionnaire started with the following question “Have you studied an Entrepreneurship course”, the answer can be only “Yes” or “No”. The students selecting “Yes” moved on to the next section, however, students saying “No” were not required to attempt further.

Data has been collected in 4 months duration between June to September 2020. The convenience sampling technique has been used to target the desired respondents. A total of 872 questionnaires were sent to respondents through online mediums. The questionnaire was developed on google docs and the link was shared through different students’ online groups on Facebook and WhatsApp, out of which 420 complete responses were received with a response rate of 48.16%. The entire data set is available in Supplementary Materials (Table S1). As regards to demographic characteristics of the respondents, the majority of them were male (*n* = 280, 66.66%). In terms of age, the majority of them belong to the age category of 25 or less (*n* = 260, 61.90%). A good number of respondents hold a bachelor’s degree (*n* = 159, 37.85%), followed by master’s degree holders (*n* = 149, 35.47%).

### 3.2. Measures

This study has adapted the items from the previously established scales. A pre-test questionnaire was implemented on 40 respondents. Further, the questionnaire was refined after consultation with three academic experts. The first part of the questionnaire was related to the items of the constructs, the second part was related to the demographics of the respondents. Each construct of the first part was measured with a five-point Likert scale ranging from strongly agree (1) to strongly disagree (5), and (3) being neutral.

#### 3.2.1. Entrepreneurship Education

The four-item measurement scale for entrepreneurial education has been adopted from the study of Walter and Block [91]. The scale intended to measure the role of teaching entrepreneurship in developing green entrepreneurial attitude, interest, and capabilities, sample item is “My school education helped me to better understand the role of entrepreneurs in society.”

#### 3.2.2. Commitment to the Environment

Alcock’s [75] seven-item scale was used to measure commitment to the environment. The scale measured the level of commitment of students to act pro-environmentally, sample items include “I am environmentally friendly in most things that I do.”

#### 3.2.3. University Green Entrepreneurial Support

University green entrepreneurial support was measured by using a four-item scale adopted from the study of Saeed et al. [78]. The university’s green entrepreneurial support has been gauged by asking questions related to green entrepreneurial resources being provided by the university. Sample items include “My university provides students with the financial and policies means to start a new business.”

#### 3.2.4. Entrepreneurial Motivation

Entrepreneurial Motivation was measured by using an eight-item scale developed by Taormina and Lao [92]. The scale measured the intrinsic motivation of the students to start a new business venture; the sample item is “I want to be a business owner.”

#### 3.2.5. Green Entrepreneurial Behavior

The venture creation process is comprised of a series of activities. You become more closer to venture creation as you complete one activity [93]. The construct green entrepreneurial behavior has been measured by using four-item scales adopted from the study of Kautonen et al. [94]. The scale envisioned to measure the completion of any major activity by the students for the pursuit of launching a green enterprise. This approach of gauging green entrepreneurship behavior is consistent with the study of Yi [28]. The sample items included “Started green product/service development.”

Complete measurement scales for all of the constructs have been provided in Table A1.

## 4. Results and Analysis

### 4.1. Assessing the Measurement Model

Assessing the structural equation model (SEM) involves tow-step approach [95,96]. Firstly, we conducted a confirmatory factor analysis (CFA) to assess validity of the measurement scales. The minimum threshold values for the goodness-of-fit indices are: x2/df = ≤ 3 [97]; NFI = >0.90 [98]; CFI = > 0.90 [99]; GFI = > 0.90 [98]; TLI = > 0.90 [97] and RMSEA = ≤ 0.08 [98]. The result of the initial measurement model was not satisfactory; therefore, we performed some modifications [100]. In total, nine items were removed to achieve satisfactory model’s fitness. Following are the final measurement model’s goodness of fit: (χ2/df = 2.126; RMSEA = 0.052; GFI = 0.926; TLI = 0.946; NFI = 0.919; CFI = 0.955).

We have used Fornell and Lacker [101] criterion for testing the reliability and discriminant validity of the constructs. They proposed three procedures for assessing the convergent validity of the measurement scales: first, to assess the item reliability of measures, second, to assess the composite reliability, third, to extract the average variance. Using standardized factor loading of the construct’s items, we have examined item reliability [102]. The standardized factor loadings of all underlying constructs were greater than 0.50, indicating adequate convergent validity of the latent constructs. The values of constructs’ composite reliability (CR) and Cronbach alphas (α) are greater than 0.70, indicating adequate reliability of the constructs of the measurement model as shown in Table 1. The average variance extracted (AVE) of the constructs are above the recommended threshold of 0.50 and the correlation coefficient of the constructs was less than the square root of the AVE’s, confirming discriminant validity of the constructs as shown in Table 2.

### 4.2. Hypotheses Testing Using Structural Equation Modeling (SEM)

**Figure 2:**
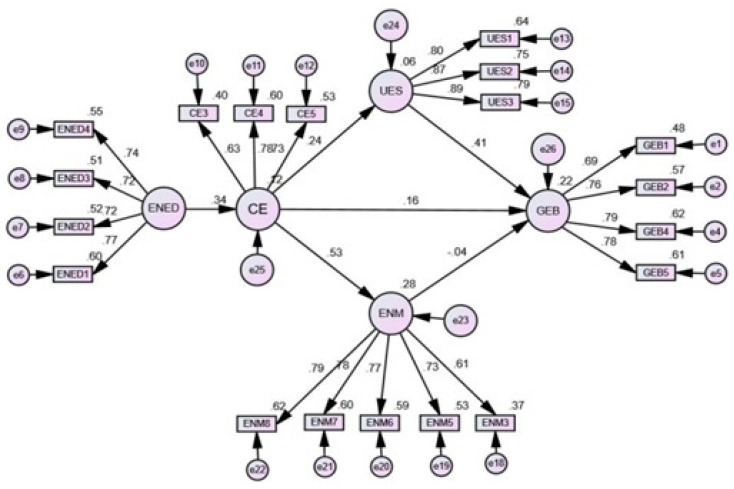
Structural Equation Model.

The structural equation modeling approach was used to assess the proposed hypotheses as shown in Figure 2. It is the most preferred method to test causal relationships [100]. Besides, this method is used to control measurement errors and test multiple relationships simultaneously [103,104]. The results of the structural model show good fit indices: x^2^/df = 2.887; GFI = 0.905; NFI = 0.887; TLI = 0.909; CFI = 0.922; RMSEA = 0.067 indicating an acceptable model fit [99].

The hypotheses testing of this study is based on path coefficients and significance value. Path coefficient values range between −1 and +1, the value closer to +1 indicates a strong relationship, and vice versa [105], and the significance value below 0.05 refers to the acceptance of hypotheses. Eight hypotheses were tested based on green entrepreneurial behavior. Hypothesis 1 proposed a significant positive influence of entrepreneurial education on the commitment to the environment was supported (β = 0.341, *p* < 0.000); hypothesis 2 proposed significant positive influence of commitment to the environment on university green entrepreneurial support was supported (β = 0.241, *p* < 0.000); hypothesis 3 proposed a significant positive influence of commitment to the environment on entrepreneurial motivation was supported (β = 0.530, *p* < 0.000); hypothesis 4 proposed a significant positive influence of commitment to the environment on green entrepreneurial behavior was supported (β = 0.164, *p* < 0.024); hypothesis 5 proposed a significant positive influence of university green support on green entrepreneurial behavior was also supported (β = 0.411, *p* < 0. 000); hypothesis 6 proposed a significant positive influence of entrepreneurial motivation on green entrepreneurial behavior was rejected (β = −0.040, *p* < 0.548).

## 5. Discussions, Implications, Limitations, and Future Research

The present study aims to present a conceptual model of green entrepreneurial behavior among university students. The conceptual model of the study comprised of potentially significant constructs that have a vital role in shaping individual behavior for entrepreneurial activity. All the hypotheses were accepted in the proposed model, except the positive influence of entrepreneurial motivation on green entrepreneurial behavior as presented in Table 3. Further, the study depicts that entrepreneurial education, commitment to the environment and university environmental support programs have a significant impact on green entrepreneurial behavior among university students.

In terms of the positive effect of entrepreneurial education on the commitment to the environment, the results confirm the relationship between entrepreneurial education and commitment to the environment, and it matches with the findings of Alam et al. [63]. This signifies that entrepreneurial education significantly affects people’s commitment towards environmental protection. For the relationship between environmental commitment and university environmental support program, the results confirmed the positive effect of environmental commitment in shaping university green support programs. For the relationship between commitment to the environment and environmental motivation, the results show that commitment to the environment significantly affects motivation to protect the environment, and this is in line with the views presented by Davis, Green, and Reed [64]. In terms of the positive effect of commitment to the environment on green entrepreneurial behavior, the findings of the study support the proposed relationship and match with the findings of Suasana and Ekawati [73], where authors argued that environmental commitment lead towards green products innovation. This depicts that individual commitment to environment has a vibrant role in shaping green entrepreneurial behavior. In terms of the positive effect of university green entrepreneurial support, the results of the study reveal that university entrepreneurial support affects the green entrepreneurial behavior of the students. The results match with the findings of previous studies where the authors have verified the effective role of universities in nurturing entrepreneurial skills among the students [76,78,83].

Prior research has shown that the flow theory is a useful construct to understand the impact of user cognition on computer-mediated technologies [52,54,106,107]. As an example, Webster, Trevino, and Ryan [107] used flow theory to explain the experience of subjective human and computer-mediated communication (CMC) technology interaction. They considered that such interaction is playful and exploratory experience. Later, Novak, Hoffman, and Yung [52] applied flow theory to comprehend customer navigation behavior in online environments such as the World Wide Web. The results of all of the above-mentioned studies contradict the results of the current study.

The conflicting results suggest that environmental motivation may not lead to launching green ventures. This can be because many other factors affect an individual in deciding his/her career path. These factors can become a cause for a particular behavior in various situations [108]. Similarly, there is a need to identify any other major factor linked with the flow theory that can significantly trigger the green entrepreneurial instinct. The lack of students’ motivation may be due to income constraints and the availability of worthwhile government jobs.

### 5.1. Contributions & Implications

The current study has multiple contributions to the existing literature of green entrepreneurship. Firstly, it integrated the resource-based view and flow theory to analyze the green entrepreneurship behavior of university students, and this is by in large an important area and has not been extensively studied in connection with entrepreneurship education and green entrepreneurship behavior [32]. Recent studies reveal that students have an inclination to become entrepreneurs but lack of resources and support hinders them to pursue it as a career [109]. Therefore, the support of educational institutions has a vital role in the promotion and adoption of green businesses. The universities can assist students through awareness programs and in the development of ideas and concepts of green businesses. We, therefore, expect that the university’s green entrepreneurial support system and entrepreneurial education are fundamental external resources that cultivate green entrepreneurial behavior. Green entrepreneurship behavior is analyzed by paying attention to university students’ commitment to environment and environmental motivation as past studies have not considered such a combination. The reason to include commitment to environment is to have a comprehensive understanding of the young generation’s attachment with environmental values. It has been demonstrated that the resource-based view perfectly contributes towards university student’s green entrepreneurial instinct. Davis, Bagozzi, and Warshaw [53] posited that intrinsic motivation can be translated into a relevant behavior by the encountering individual. However, the current study presents a conflicting viewpoint and it is suggested that flow theory must be combined with any other relevant factor to evoke entrepreneurial drive amongst university students.

This study has also multiple practical implications. First, the study depicts that entrepreneurial education leads to a commitment to the environment. This provides insights regarding the effectiveness of entrepreneurial education in environmental sustainability. Therefore, academicians and practitioners should focus on entrepreneurial education focusing on environmental benefits to the general public. Second, the significant effect of commitment to the environment on university environmental support, environmental motivation, and green entrepreneurial behavior demonstrates the effectiveness of individual commitment towards sustainability. Individual commitment to the environment signifies that it can change the attitude of the university towards environmental courses/program offerings, increasing motivation to preserve the environment and support green entrepreneurial activity. Third, university environmental support has an impact on green entrepreneurial behavior illustrates the importance of university environmental programs in shaping the behavior towards green entrepreneurship. Therefore, policymakers and academicians should design entrepreneurship courses in such a manner that incorporate environmental issues and promote sustainability. For, example, previous studies suggest that entrepreneurial skills can be acquired through practical experiences [57,110,111]. Additionally, we suggest that academicians in Pakistan should not only design entrepreneurship courses but also on how to inspire students to launch green ventures.

### 5.2. Limitations and Future Research

The current research is focused on the resource-based view and flow theory. Resources play a pivotal role in launching and operating a business venture. This study aimed at linking the resource-based view with the green entrepreneurial behavior, which has been supported. Several resources have been identified by the theorists e.g., [41,42,43]. However, we have only used entrepreneurship education, commitment to environment, university green entrepreneurial support, and environmental motivation as the predictors of green entrepreneurship behavior, future researches can be done by incorporating other institutional and structural factors. The literature from Flow theory has been adopted in the study to find out the effect of internal motivation to launch green enterprises. Surprisingly it has not been supported by the data. This shows that motivation alone is not a deciding factor for launching a green business. Aspiring students must be given the proper platform to work for environmental sustainability. Green entrepreneurship behavior has been gauged by taking into account any major activity performed towards the launch of green enterprise, as suggested by experts [94,95].

The study mainly applied a cross-sectional approach in collecting data, the long term environmentally sustainable behavior can be further dug out by following a longitudinal approach. Furthermore, green entrepreneurship behavior has been studied in general, in future researches specific environmentally friendly enterprises can be studied and how they are influencing society. This study ends at identifying the behavior, it is strongly recommended that a study must be undertaken to find out the impact of launching such business enterprises. These results can be attained by applying the previously discussed longitudinal study design.

## 6. Conclusions

The literature of entrepreneurship provides ample evidence regarding the role of entrepreneurial education in green entrepreneurial behavior, but it demands thorough investigation into its factors. This study academically fills the theoretical gap and suggests policy implications for the strategists and academicians. The present study has considered entrepreneurial education, commitment to the environment, university green environmental support, and environmental motivation as antecedents of green entrepreneurial behavior.

The results depict that entrepreneurial education has a significant impact on the commitment to the environment. This validated the effective role of entrepreneurial education in the proposed model. Theoretically, the addition of commitment to the environment in the conceptual model has contributed to green entrepreneurship literature. Commitment to the environment is a crucial factor as it has a significant influence on student’s environmental motivation, university green environmental support, and green entrepreneurial behavior. Further, this study incorporated the effectiveness of resources based view by using university green entrepreneurial support programs into the model. This has been successfully supported by empirical evidence. University support has been well studied by previous researchers around the globe [76,77,78]. However, studies have paid little attention to university green entrepreneurial support, which is a crucial antecedent affecting green entrepreneurial behavior.

## Figures and Tables

**Table 1:** Descriptive analysis and measurement model.

Constructs Items Standardized Cronbach’s Alpha Factor Loading (ά)			Convergent Validity	
			Composite Reliability (CR)	Average Variance Extracted (AVE)
Entrepreneurship Education	ENED1	0.77		
	ENED2	0.71 0.823	0.827	0.545
	ENED3	0.71		
	ENED4	0.76		
University Green Entrepreneurial Support	UES1	0.80		
	UES2	0.87 0.886	0.890	0.730
	UES3	0.89		
Commitment to Environment	CE3	0.64		
	CE4	0.79 0.754	0.761	0.517
	CE5	0.72		
Entrepreneurial Motivation	ENM3	0.61		
	ENM5	0.73 0.851	0.794	0.564
	ENM6	0.77		
	ENM7	0.77		
	ENM8	0.79		
Green Entrepreneurial Behavior	GEB1	0.69		
	GEB2	0.76 0.842	0.842	0.572
	GEB4	0.79		
	GEB5	0.78		

**Note:** ENED = Entrepreneurial Education, UES = University Green Entrepreneurial Support, CE = Commitment to Environment, ENM = Entrepreneurial Motivation, GEB = Green Entrepreneurial Behavior.

**Table 2:** Discriminant validity of constructs.

Latent Variables	1	2	3	4	5
Entrepreneurship Education	**0.738**				
University Green Entrepreneurial Support	0.499	**0.854**			
Commitment to Environment	0.226	0.168	**0.719**		
Entrepreneurial Motivation	0.229	0.077	0.435	**0.736**	
Green Entrepreneurial Behavior	0.291	0.405	0.167	0.063	**0.756**

**Note:** The diagonals (in bolds) represent the square root of AVE, and off-diagonal values represent the correlations of each construct with other constructs.

**Table 3:** Hypotheses assessment summary.

Hypotheses	Beta	SE	*p*-Values	*t*-Values	Decision
Hypothesis 1 (H1): ENED → CE	0.341	0.052	0.000 **	5.318	Accepted
Hypothesis 2 (H2): CE → UES	0.241	0.109	0.000 **	4.017	Accepted
Hypothesis 3 (H3): CE → ENM	0.530	0.059	0.000 **	7.194	Accepted
Hypothesis 4 (H4): CE → GEB	0.164	0.103	0.024 *	2.255	Accepted
Hypothesis 5 (H5): UES → GEB	0.411	0.046	0.000 **	6.905	Accepted
Hypothesis 6 (H6): ENM → GEB	−0.040	0.117	0.548	−0.601	Rejected

**Note:** Path coefficients (Beta); *t*-values for one-tailed; 2.33 (*p* < 0.01 **), 1.645 (*p* < 0.05 *). *t*-values for two-tailed; 2.58 (*p* < 0.01 **), 1.96 (*p* < 0.05 *).

## Data Availability

The data presented in this article are available on request from the corresponding author. The data are not publicly available due to privacy.

## Supplementary Materials

The following are available online at https://www.mdpi.com/1660-4601/18/2/550/s1, Table S1: Data set.

## Appendix A

**Table A1:** Measurement scales.

Construct	Items
Entrepreneurial Education:	ENED 1: My school education helped me develop my sense of initiative—a sort of entrepreneurial attitude.
	ENED 2: My school education helped me to better understand the role of entrepreneurs in society.
	ENED 3: My school education made me interested to become an entrepreneur.
	ENED 4: My school education gave me skills and know-how that enable me to run a business.
Commitment to Environment:	CE 1: It takes too much time and effort to do things that are environmentally friendly.
	CE 2: Scientists will find a solution to global warming without people having to make big changes to their lifestyle.
	CE 3: The environment is a low priority for me compared with a lot of other things in my life.
	CE 4: I am environmentally friendly in most things that I do.
	CE 5: Most people in Pakistan today need to change their way of life so that future generations can continue to enjoy a good quality of life and environment.
	CE 6: I personally need to change my way of life so that future generations can continue to enjoy a good quality of life and environment.
	CE 7: I frequently feel the need to reduce carbon emissions which affect what I do, for example by choosing to drive less or to turn lights off when I can.
University Green Entrepreneurial Support:	UES1 My university offers courses on green entrepreneurship.
	UES2 My university motivates students to start a green business.
	UES3 My university offers project work focused on green entrepreneurship.
	UES4 My university provides students the financial and policy-related advice to start a new business.
Entrepreneurial Motivation:	How much do you agree that the following statements describe you?
	ENM 1: I want to be a business owner.
	ENM 2: I want to profit from my endeavors.
	ENM 3: I like to be told how to do my job.
	ENM 4: I enjoy having authority at work.
	ENM 5: I like to control my own time at work.
	ENM 6: I think that having a business can improve my financial status.
	ENM 7: I see a good future for myself if I start a business.
	ENM 8: I like to make business decisions.
Green Entrepreneurial Behavior:	GEB 1: Written a green business plan.
	GEB 2: Started green product/service development.
	GEB 3: Attempted to obtain external funding.
	GEB 4: Purchased material, equipment or machinery for the green business.
	GEB 5: Registered the green company.

## References

[1] Kickul J., Lyons T.S. (2020). Understanding Social Entrepreneurship: The Relentless Pursuit of Mission in an Ever Changing World.

[2] Beazley M. (1993). Caring for the Earth: A Strategy for Survival.

[3] Waris I., Hameed I. An empirical study of consumers intention to purchase energy efficient appliances. Soc. Responsib. J..

[4] Hameed I., Khan K. (2020). An extension of the goal-framing theory to predict consumer’s sustainable behavior for home appliances. Energy Effic..

[5] (2017). Pakistan Climate Change Act. http://www.na.gov.pk/uploads/documents/1491459994_555.pdf.

[6] Environmental Performance Index. https://epi.envirocenter.yale.edu/.

[7] (2019). The Clean Green Pakistan Index. https://cleangreen.gov.pk/implementation-approach.

[8] (2019). National Youth Development Framework. https://www.app.com.pk/national/pti-govt-devises-national-youth-development-framework-empowering-youth/.

[9] (2011). United Nation Environment Programme. https://www.unenvironment.org/resources/annual-report/unep-2011-annual-report.

[10] (2020). National Incubation Center. https://nicpakistan.pk/.

[11] (2019). Pakistan Economic Survey. http://www.finance.gov.pk/survey_1920.html.

[12] (2018). Pakistan Bureau of Statistics. http://www.pbs.gov.pk/content/pakistan-employment-trends-2018.

[13] Borasi R., Finnigan K. (2010). Entrepreneurial Attitudes and Behaviors that Can Help Prepare Successful Change-Agents in Education. New Educ..

[14] Malen J., Marcus A.A. (2017). Promoting clean energy technology entrepreneurship: The role of external context. Energy Policy.

[15] York J.G., Venkataraman S. (2010). The entrepreneur–environment nexus: Uncertainty, innovation, and allocation. J. Bus. Ventur..

[16] Harju J., Kosonen T. (2012). The Impact of Tax Incentives on the Economic Activity of Entrepreneurs.

[17] Koe W.-L., Omar R., Sa’Ari J.R. (2015). Factors Influencing Propensity to Sustainable Entrepreneurship of SMEs in Malaysia. Procedia Soc. Behav. Sci..

[18] Omar R., Samuel R. (2011). Environmental Management Amongst Manufacturing Firms in Malaysia. Proceedings of the 3rd International Symposium & Exhibition in Sustainable Energy & Environment (ISESEE).

[19] Rutherfoord R., Blackburn R., Spence L. (2000). Environmental management and the small firm: An international comparison. Int. J. Entrep. Behav. Res..

[20] (2002). Environment Agency NetRegs Benchmarking Survey: A Snapshot of Environmental Awareness and Practice in Small and Medium Sized Enterprises (SMEs). www.environment-agency.gov.uk/netregs.

[21] Hameed I., Haq M.A. Book Review: Research, Innovation and Entrepreneurship in Saudi Arabia: Vision 2030. Int. Small Bus. J..

[22] Hameed I., Hyder Z., Imran M., Shafiq K. Greenwash and green purchase behavior: An environmentally sustainable perspective. Environ. Dev. Sustain..

[23] Sedlacek S. (2013). The role of universities in fostering sustainable development at the regional level. J. Clean. Prod..

[24] Bae T.J., Qian S., Miao C., Fiet J.O. (2014). The Relationship Between Entrepreneurship Education and Entrepreneurial Intentions: A Meta-Analytic Review. Entrep. Theory Pr..

[25] Martin B.C., McNally J.J., Kay M.J. (2013). Examining the formation of human capital in entrepreneurship: A meta-analysis of entrepreneurship education outcomes. J. Bus. Ventur..

[26] Block J.H., Hoogerheide L., Thurik R. (2013). Education and entrepreneurial choice: An instrumental variables analysis. Int. Small Bus. J..

[27] Souitaris V., Zerbinati S., Al-Laham A. (2007). Do entrepreneurship programmes raise entrepreneurial intention of science and engineering students? The effect of learning, inspiration and resources. J. Bus. Ventur..

[28] Walter S.G., Dohse D. (2012). Why mode and regional context matter for entrepreneurship education. Entrep. Reg. Dev..

[29] (2020). Higher Education Commission. https://www.hec.gov.pk/english/universities/pages/recognised.aspx.

[30] Klofsten M., Fayolle A., Guerrero M., Mian S., Urbano D., Wright M. (2019). The entrepreneurial university as driver for economic growth and social change—Key strategic challenges. Technol. Forecast. Soc. Chang..

[31] Findler F., Schönherr N., Lozano F.J., Reider D., Martinuzzi A. (2019). The impacts of higher education institutions on sustainable development. Int. J. Sustain. High. Educ..

[32] Yi G. (2020). From green entrepreneurial intentions to green entrepreneurial behaviors: The role of university entrepreneurial support and external institutional support. Int. Entrep. Manag. J..

[33] Teo T., Zhou M., Fan A.C.W., Huang F. (2019). Factors that influence university students’ intention to use Moodle: A study in Macau. Educ. Technol. Res. Dev..

[34] Ginanjar A. (2016). Entrepreneurship Education and Entrepreneurial Intention on Entrepreneurship Behavior: A Case Study. Proceedings of the 2016 Global Conference on Business, Management and Entrepreneurship.

[35] Demirel P., Li Q.C., Rentocchini F., Tamvada J.P. (2019). Born to be green: New insights into the economics and management of green entrepreneurship. Small Bus. Econ..

[36] Matlay H., Packham G., Jones P., Miller C., Pickernell D., Thomas B. (2010). Attitudes towards entrepreneurship education: A comparative analysis. Educ. Train..

[37] Oosterbeek H., Van Praag M., Ijsselstein A. (2010). The impact of entrepreneurship education on entrepreneurship skills and motivation. Eur. Econ. Rev..

[38] von Graevenitz G., Harhoff D., Weber R. (2010). The effects of entrepreneurship education. J. Econ. Behav. Org..

[39] Lotfi M., Yousefi A., Jafari S. (2018). The Effect of Emerging Green Market on Green Entrepreneurship and Sustainable Development in Knowledge-Based Companies. Sustainability.

[40] Penrose E.T. (1959). The Theory of the Growth of the Firm.

[41] Barney J. (1991). Firm Resources and Sustained Competitive Advantage. J. Manag..

[42] Fichter K., Tiemann I. (2018). Factors influencing university support for sustainable entrepreneurship: Insights from explorative case studies. J. Clean. Prod..

[43] Wernerfelt B. (1984). A resource-based view of the firm. Strat. Manag. J..

[44] Alvarez S.A., Busenitz L.W. (2001). The entrepreneurship of resource-based theory. J. Manag..

[45] Timmons J.A. (1999). New Venture Creation: Entrepreneurship in the 21th Centuries.

[46] Hameed I., Khan M.B., Shahab A., Hameed I., Qadeer F. (2016). Science, Technology and Innovation through Entrepreneurship Education in the United Arab Emirates (UAE). Sustainability.

[47] Hameed I., Irfan Z. (2019). Entrepreneurship education: A review of challenges, characteristics and opportunities. Entrep. Educ..

[48] Csikszentmihalyi M. (1975). Beyond Boredom and Anxiety.

[49] Csikszentmihalyi M. (1990). Flow: The Psychology of Optimal Experience.

[50] Liao L. (2006). A Flow Theory Perspective on Learner Motivation and Behavior in Distance Education. Distance Educ..

[51] Koufaris M. (2002). Applying the Technology Acceptance Model and Flow Theory to Online Consumer Behavior. Inf. Syst. Res..

[52] Novak T.P., Hoffman D.L., Yung Y.-F. (2000). Measuring the Customer Experience in Online Environments: A Structural Modeling Approach. Mark. Sci..

[53] Davis F.D., Bagozzi R.P., Warshaw P.R. (1992). Extrinsic and Intrinsic Motivation to Use Computers in the Workplace. J. Appl. Soc. Psychol..

[54] Trevino L.K., Webster J. (1992). Flow in computer-mediated communication: Electronic mail and voice mail evaluation and impacts. Comm. Res..

[55] Chan T.S., Ahern T.C. (1999). Targeting motivation—Adapting flow theory to instructional design. J. Educ. Comp. Res..

[56] Shi L., Yao X., Wu W. (2019). Perceived university support, entrepreneurial self-efficacy, heterogeneous entrepreneurial intentions in entrepreneurship education: The moderating role of the Chinese sense of face. J. Entrep. Emerg. Econ..

[57] Mustafa M.J., Hernandez E., Mahon C., Chee L.K. (2016). Entrepreneurial intentions of university students in an emerging economy. J. Entrep. Emerg. Econ..

[58] Waris I., Farooq M., Hameed I., Shahab A. (2020). Promoting sustainable ventures among university students in Pakistan: An empirical study based on the theory of planned behavior.

[59] Henderson R., Robertson M. (2000). Who wants to be an entrepreneur? Young adult attitudes to entrepreneurship as a career. Career Dev. Int..

[60] Wals A.E., The United Nations Educational, Scientific and Cultural Organization (2009). Review of Contexts and Structures for Education for Sustainable Development: 2009.

[61] Liñán F., Fayolle A. (2015). A systematic literature review on entrepreneurial intentions: Citation, thematic analyses, and research agenda. Int. Entrep. Manag. J..

[62] Beynaghi A., Trencher G., Moztarzadeh F., Mozafari M., Maknoon R., Filho W.L. (2016). Future sustainability scenarios for universities: Moving beyond the United Nations Decade of Education for Sustainable Development. J. Clean. Prod..

[63] Alam M.Z., Kousar S., Rehman C.A. (2019). Role of entrepreneurial motivation on entrepreneurial intentions and behaviour: Theory of planned behaviour extension on engineering students in Pakistan. J. Glob. Entrep. Res..

[64] Davis J.L., Green J.D., Reed A. (2009). Interdependence with the environment: Commitment, interconnectedness, and environmental behavior. J. Environ. Psychol..

[65] Ekawati N.W., Kertiyasa N.N., Giantari G.A.K., Sariyathi N.K. (2017). Ecopreneurship and Green Innovation for the Success of New Spa Products. J. Bus. Retail. Manag. Res..

[66] Robichaud Y., McGraw E., Alain R. (2001). Toward the development of a measuring instrument for entrepreneurial motivation. J. Dev. Entrep..

[67] Wiklund J., Shepherd D. (2003). Knowledge-based resources, entrepreneurial orientation, and the performance of small and medium-sized businesses. Strat. Manag. J..

[68] Preedy M., Glatter R., Levačić R. (1997). Educational Management: Strategy, Quality, and Resources.

[69] Erçetin Ş.Ş. (2016). Applied Chaos and Complexity Theory in Education.

[70] Shen Y. (2008). The Effect of Changes and Innovation on Educational Improvement. Int. Edu. Stud..

[71] Matoti S.N. (2010). The unheard voices of educators: Perceptions of educators about the state of education in South Africa. S. Afr. J. High. Edu..

[72] Chedli E.P., Kchaich M. (2016). Entrepreneurial Motivation And Performance Of Enterprises. Econ. Manag. Fin. Mark..

[73] Suasana I.G.A.K.G., Ekawati N.W. (2018). Environmental commitment and green innovation reaching success new products of creative industry in Bali. J. Bus. Retail. Manag. Res..

[74] Delmar F., Wiklund J. (2008). The Effect of Small Business Managers’ Growth Motivation on Firm Growth: A Longitudinal Study. Entrep. Theory Pr..

[75] Alcock I. (2012). Measuring Commitment to Environmental Sustainability: The Development of a Valid and Reliable Measure. Methodol. Innov. Online.

[76] Bergmann H., Hundt C., Sternberg R. (2016). What makes student entrepreneurs? On the relevance (and irrelevance) of the university and the regional context for student start-ups. Small Bus. Econ..

[77] Etzkowitz H. (2004). The evolution of the entrepreneurial university. Int. J. Technol. Glob..

[78] Saeed S., Yousafzai S.Y., Yani-De-Soriano M., Muffatto M. (2015). The Role of Perceived University Support in the Formation of Students’ Entrepreneurial Intention. J. Small Bus. Manag..

[79] Wagner M., Schaltegger S., Hansen E.G., Fichter K. (2019). University-linked programmes for sustainable entrepreneurship and regional development: How and with what impact?. Small Bus. Econ..

[80] Ploum L., Blok V., Lans T., Omta O. (2018). Toward a Validated Competence Framework for Sustainable Entrepreneurship. Organ. Environ..

[81] Rideout E.C., Gray D.O. (2013). Does Entrepreneurship Education Really Work? A Review and Methodological Critique of the Empirical Literature on the Effects of University-Based Entrepreneurship Education. J. Small Bus. Manag..

[82] Duval-Couetil N. (2013). Assessing the Impact of Entrepreneurship Education Programs: Challenges and Approaches. J. Small Bus. Manag..

[83] Karimi S., Biemans H.J.A., Lans T., Chizari M., Mulder M. (2016). The Impact of Entrepreneurship Education: A Study of Iranian Students’ Entrepreneurial Intentions and Opportunity Identification. J. Small Bus. Manag..

[84] Jwara N., Hoque M. (2018). Entrepreneurial intentions among university students: A case study of Durban University of Technology. Acad. Entrep. J..

[85] Krueger N.F., Reilly M.D., Carsrud A.L. (2000). Competing models of entrepreneurial intentions. J. Bus. Ventur..

[86] Collins C.J., Hanges P.J., Locke E.A. (2004). The Relationship of Achievement Motivation to Entrepreneurial Behavior: A Meta-Analysis. Hum. Perform..

[87] Van der Zwan P., Verheul I., Thurik A.R. (2012). The entrepreneurial ladder, gender, and regional development. Small Bus. Econ..

[88] Rekha S.K., Ramesh S., Jaya-Bharathi S. (2015). Empirical study onthe relationship between entrepreneurial mindset and the factors affect-ing intrapreneurship: A study in Indian context. Int. J. Entrep..

[89] Townsend D.M., Busenitz L.W., Arthurs J.D. (2010). To start or not to start: Outcome and ability expectations in the decision to start a new venture. J. Bus. Ventur..

[90] McMullan W.E., Long W.A., Wilson A. (1985). MBA concentration on entrepreneurship. J. Small Bus. Entrep..

[91] Walter S.G., Block J.H. (2016). Outcomes of entrepreneurship education: An institutional perspective. J. Bus. Ventur..

[92] Taormina R.J., Lao S.K.M. (2007). Measuring Chinese entrepreneurial motivation. Int. J. Entrep. Behav. Res..

[93] Van Gelderen M., Kautonen T., Fink M. (2015). From entrepreneurial intentions to actions: Self-control and action-related doubt, fear, and aversion. J. Bus. Ventur..

[94] Kautonen T., Hatak I., Kibler E., Wainwright T. (2015). Emergence of entrepreneurial behaviour: The role of age-based self-image. J. Econ. Psychol..

[95] Zaman U., Jabbar Z., Nawaz S., Abbas M. (2019). Understanding the soft side of software projects: An empirical study on the interactive effects of social skills and political skills on complexity–performance relationship. Int. J. Project Manage..

[96] Anderson J.C., Gerbing D.W. (1988). Structural equation modeling in practice: A review and recommended two-step approach. Psych. Bull..

[97] Schreiber J.B., Nora A., Stage F.K., Barlow E.A., King J. (2006). Reporting Structural Equation Modeling and Confirmatory Factor Analysis Results: A Review. J. Educ. Res..

[98] Hu L.T., Bentler P.M. (1999). Cutoff criteria for fit indexes in covariance structure analysis: Conventional criteria versus new alternatives. Struct. Equ. Model..

[99] Hooper D., Coughlan J., Mullen M. Evaluating model fit: A synthesis of the structural equation modelling literature. Proceedings of the 7th European Conference on Research Methodology for Business and Management Studies.

[100] Bagozzi R., Yi Y. (2012). Specification, evaluation, and interpretation of structural equation models. J. Acad. Mark. Sci..

[101] Fornell C., Larcker D.F. (1981). Evaluating structural equation models with unobservable variables and measurement error. J. Mark. Res..

[102] Zaman U., Nawaz S., Tariq S., Humayoun A.A. (2019). Linking transformational leadership and “multi-dimensions” of project success: Moderating effects of project flexibility and project visibility using PLS-SEM. Int. J. Manag. Projects Bus..

[103] Byrne B.M. (2013). Structural Equation Modeling with Mplus: Basic Concepts, Applications, and Programming.

[104] Zaman U. (2020). Examining the effect of xenophobia on “transnational” mega construction project (MCP) success: Moderating role of transformational leadership and high-performance work (HPW) practices. Eng. Const. Archit Manage..

[105] Hair J.F., Ringle C.M., Sarstedt M. (2011). PLS-SEM: Indeed a Silver Bullet. J. Mark. Theory Pr..

[106] Ghani J.A., Deshpande S.P. (1994). Task Characteristics and the Experience of Optimal Flow in Human—Computer Interaction. J. Psychol..

[107] Webster J., Trevino L.K., Ryan L. (1993). The dimensionality and correlates of flow in human-computer interactions. Comput. Hum. Behav..

[108] Pearl R.L., Dovidio J.F., Puhl R.M., Brownell K.D. (2015). Exposure to Weight-Stigmatizing Media: Effects on Exercise Intentions, Motivation, and Behavior. J. Health Commun..

[109] Asante E.A., Affum-Osei E. (2019). Entrepreneurship as a career choice: The impact of locus of control on aspiring entrepreneurs’ opportunity recognition. J. Bus. Res..

[110] Rodriguez-Gutierrez P., Cabeza-Ramírez L.J., Muñoz-Fernández G.A. (2020). University Students’ Behaviour towards Entrepreneurial Intention in Ecuador: Testing for the Influence of Gender. Int. J. Environ. Res. Public Health.

[111] Arias-Aranda D., Bustinza-Sánchez O. (2009). Entrepreneurial attitude and conlict management through business simulations. Ind. Manag. Data Syst..

